# Factors affecting time to sputum culture conversion and treatment outcome of patients with multidrug-resistant tuberculosis in China

**DOI:** 10.1186/s12879-018-3021-0

**Published:** 2018-03-06

**Authors:** Qiao Liu, Peng Lu, Leonardo Martinez, Haitao Yang, Wei Lu, Xiaoyan Ding, Limei Zhu

**Affiliations:** 1 Department of Chronic Communicable Disease, Center for Disease Control and Prevention of Jiangsu Province, Nanjing, Jiangsu Province People’s Republic of China; 2 Department of Epidemiology and Biostatistics, School of Public Health, Nanjing Medical University, Nanjing, People’s Republic of China; 3 Department of Epidemiology and Biostatistics, University of Georgia School of Public Health, Athens, Georgia; 4 Institute of Parasitic Disease of Jiangsu Province, Wuxi, Jiangsu Province People’s Republic of China

**Keywords:** Multidrug-resistant tuberculosis, Risk factors, Sputum culture conversion, Treatment outcome

## Abstract

**Background:**

Few prospective cohort studies, none in China, have investigated the relationship between treatment outcomes of multidrug-resistant tuberculosis (MDR-TB) patients and sputum culture conversion. Factors affecting the time of the culture conversion throughout the whole course of the treatment have rarely been investigated.

**Methods:**

This study was performed in four cities in Jiangsu province, China. MDR-TB patients were consecutively enrolled between December 2011 and March 2014. Rates of sputum culture conversion were calculated and Cox proportional-hazards model was performed. Factors contributing to sputum culture conversion were investigated.

**Results:**

In all, 139 MDR-TB patients with treatment outcomes were enrolled. Median time to culture conversion among those who converted was 91.5 days (interquartile range, 34.0–110.8 days). After multivariable analysis, smoking (HR = 0.44; 95% CI: 0.23–0.83), drinking (HR = 0.41; 95% CI: 0.21–0.81), ofloxacin resistance (HR = 0.43; 95% CI: 0.24–0.76) and sputum smear grade > 1 (HR = 0.51; 95% CI: 0.31–0.83) were less likely to have culture conversion.

**Conclusions:**

MDR-TB patients who smoke, drink, have ofloxacin resistance, or a high smear grade are less likely to respond to treatment and should be meticulously followed up.

## Background

Tuberculosis (TB) now exceeds human immunodeficiency virus (HIV) as the infectious disease responsible for the greatest number of deaths globally [1]. In addition, multidrug-resistant tuberculosis (MDR-TB), patients resistant to rifampicin (INH) and isoniazid (RMP), hampers the prevention and control of tuberculosis. Due to poor effectiveness and elevated costs in treatment of MDR-TB patients, they may endure longer infectious periods than those with drug-susceptible TB [2]. Early identification and diagnosis of MDR-TB patients are essential to further prevent the spread of MDR-TB and provide comprehensive and successful treatment.

Sputum culture plays an important role in monitoring treatment response in MDR-TB patients [3]. Sputum culture conversion is a clinical tool used to predict therapeutic efficacy in MDR-TB patients. Non-conversion of sputum culture at the end of the intensive phase of treatment tends to yield unfavorable outcomes, more specifically with failure and death [4, 5].

Evidence exists suggesting that sputum culture conversion after the first 2 months may be an early predictor of treatment success in MDR-TB patients [6–8]. Lung cavitation at baseline chest X-ray and resistance to ofloxacin (Ofx) or streptomycin have been associated with a delay in sputum culture conversion in prior studies [6]. Although sputum culture conversion has been used substantially in clinical settings, few prospective cohort studies have been performed in China and India, two countries with almost half of the global burden of drug resistant tuberculosis [9, 10]. In addition, factors affecting the time of the culture conversion throughout the whole course of the treatment have rarely been investigated.

Through a prospective cohort study design in urban China, we analyzed secondary factors that influenced sputum culture conversion in the process of treatment of MDR-TB disease.

## Methods

### Study design and patients

A prospective cohort study was conducted in four cities (Xuzhou, Lianyungang, Zhenjiang, and Nantong) in Jiangsu province, China, as previously described [11]. All MDR-TB patients were consecutively enrolled in the study between December 2011 and March 2014. MDR-TB patients were identified at the time of diagnosis by regional reference laboratories using traditional drug sensitivity tests (DST), as previously described [12]. Patients that tested sputum culture negative at baseline were excluded. Once confirmed, every patient signed an informed consent form, after which a questionnaire designed by local investigators was administered to gather important demographic and clinical information. The questionnaire contained characteristics including age, sex, weight, and any history of smoking or drinking. In addition, laboratory examination information was collected including sputum smear, sputum culture, chest radiograph findings, and drug sensitivity test results. Sputum culture results were examined at monthly intervals during the intensive phase and 2-monthly intervals during the continuation phase.

### Definitions

World Health Organization guidelines [13] were consulted when defining individuals and variables in our study. MDR-TB patients were defined as those who were resistant to at least INH and RMP for *Mycobacterium tuberculosis* (MTB) in vitro. A positive sputum culture was defined as a colony for MTB [5] and negative when there was no acid-fast bacillus (AFB) in 300 fields. Sputum culture conversion was defined as two consecutive negative cultures after the first sputum sample, collected at least 30 days apart. Persistent positive sputum culture was defined as no sputum culture conversion during the two years treatment period. Final outcomes included success, failure, and death. Sputum smear grading of TB patients was as follows: 1+ (3–9 AFB in 100 fields), 2+ (1–9 AFB in 10 fields), 3+ (1–9 AFB in 1 field) and 4+ (≥10 AFB in 1 field). Sputum smear grading was divided into two groups, i.e., ≤1 (includes sputum smear negative and positive 1+) and > 1 (includes sputum smear positive 2+, 3+, and 4+).

### Regimens

Directly Observed Treatment was administered by trained nurses during hospitalization or through in-house visits by a physician. The standardized regimen of patients usually contains two phase: the intensive phase and the continuation phase. The total duration of therapeutics for MDR-TB patients is 24-27 months, depending on the duration of the intensive phase [14]. Drugs used to treat MDR-TB included pyrazinamide (Z), ethambutol (E), kanamycin (Km), amikacin (Am), capreomycin (Cm), Ofx, levofloxacin (Lfx), moxifloxacin (Mfx), cycloserine (Cs), para-amino salicylic acid (PAS), and protionamide (Pto). All confirmed MDR-TB patients were treated with a standardized treatment regimen or an individualized regimen of second line drugs, devised based on a patient’s history of TB treatment and DST results.

### Statistical analysis

Statistical analysis was conducted using SPSS software (version 23.0). Cox proportional-hazards analysis was used to evaluate the hazard ratio (HR). Variables were considered suggestive of potential statistical significance if the *P*-value was < 0.05 in univariate analysis. Multivariable analysis was used to estimate HR and adjusted survival curves. Age and sex were put into the multivariable model regardless of their *P*-value< 0.05. HRs of the differences between the two groups were calculated to evaluate influential factors affecting time to sputum culture conversion. When the HR was < 1, the variable was a risk factor.

## Results

### Characteristics of enrolled MDR-TB patients

**Fig. 1:**
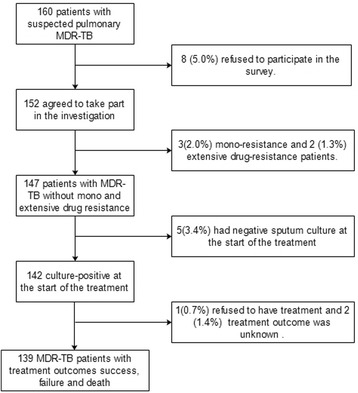
Flow diagram of multidrug-resistant patients included in this study

Between 2011 and 2014, a total of 160 suspected pulmonary MDR-TB patients were recruited of which 21 did not meet study inclusion criteria (Fig. 1). A total of 139 pulmonary MDR-TB patients with a treatment outcome classified as successful, failure, and death were enrolled in our investigation. There were no patients that started the study and then dropped out of the study.

Table 1 displays demographic and clinical characteristics of included MDR-TB patients. The median age and weight of the patients were 51 years old and 60 kg, respectively; 99 (71.4%) were male; 65 (46.8%) smoked previously or at the time of treatment, and 26 (18.7%) drank alcohol.

**Table 1:** Demographic and clinical characteristics of 139 multidrug-resistant tuberculosis patients

Variable	Median (IQR)	No. (%)
Gender		
Male		99 (71.4)
Female		40 (28.6)
Age, years	51.0 (35.8–60.0)	
≤ 51		71 (51.1)
> 51		68 (48.9)
Weight, kilograms	60.0 (53.0–65.0)	
≤ 60		79 (56.8)
> 60		60 (43.2)
Occupation		
Peasant		44 (31.7)
Worker		18 (13.0)
Unemployed		61 (43.9)
Others^&^		16 (11.5)
Smoking		
Yes		65 (46.8)
No		72 (51.8)
Missing		2 (1.4)
Drinking		
Yes		26 (18.7)
No		86 (61.9)
Missing		27 (19.4)
Lung cavitation		
Yes		72 (51.8)
No		62 (44.6)
Missing		3 (2.2)
Lung lesions		
Yes		129 (92.8)
No		3 (2.2)
Missing		7 (5.0)
Resistant to ofloxacin		
Yes		42 (58.3)
No		81 (30.2)
Missing		16 (11.5)
Resistant to Kanamycin		
Yes		15 (10.8)
No		103 (74.1)
Missing		21 (15.1)
Sputum smear grade		
≤ 1		51 (36.7)
> 1		87 (62.6)
Missing		1 (0.7)

Abbreviations: *No*. number *IQR* interquartile range. & including civil servants, teachers, students etc

Of 139 MDR-TB patients, 84 (60.4%) patients had a successful treatment outcome and 55 (39.6%) experienced either a failed treatment or died. In all, 106 (76.3%) patients had sputum-culture conversion, of which 18 had a second positive sputum culture during the first year of the treatment. Median time to culture conversion among those who converted was 91.5 days (interquartile range, 34.0–110.8 days).

### Baseline regimens

Among 139 patients, 63 (45.3%) were treated with individualized regimens (IR); 42 (30.2%) were treated with standard regimen 1 (SR1: 6Z Am Lfx Cs Pto/18Z Lfx Cs Pto); 16 (11.5%)were treated with standard regimen 2 (SR2: 6Z Am Lfx PAS Pto/18Z Lfx PAS Pto); 3 (2.2%) were treated with standard regimen 3 (SR3: 6Z Cm Lfx Cs Pto/18Z Lfx Cs Pto); 15 patients (10.8%) were treated with standard regimen 4 (SR4: 6Z Cm Lfx PAS Pto/18Z Lfx Cs Pto) (Table 2).

**Table 2:** Chemotherapy regimens of the MDR-TB patients

Variable	Chemotherapy regimen	N (%)
Individualized regimen	Distinct from patient-to-patient	63 (45.3%)
Regimen 1	6 Pyrazinamide Amikacin Levofloxacin Cycloserine Protionamide	42 (30.2%)
	/18 Pyrazinamide Levofloxacin Cycloserine Protionamide	
Regimen 2	6 Pyrazinamide Amikacin Levofloxacin Para-amino salicylic acid Protionamide	16 (11.5%)
	/18 Pyrazinamide Levofloxacin Para-amino salicylic acid Protionamide	
Regimen 3	6 Pyrazinamide Capreomycin Levofloxacin Cycloserine Protionamide	3 (2.2%)
	/18 Pyrazinamide Levofloxacin Cycloserine Protionamide	
Regimen 4	6 Pyrazinamide Capreomycin Levofloxacin Para-amino salicylic acid Protionamide	15 (10.8%)
	/18 Pyrazinamide Levofloxacin Cycloserine Protionamide	

Abbreviations: *MDR-TB* multidrug-resistant tuberculosis, *N* number

### Factors contributing to sputum culture conversion

In univariate analysis (Table 3), we found that factors associated with reduced rate of sputum culture conversion were smoking (HR = 0.63; 95% CI: 0.43–0.93; *P* = 0.020), drinking (HR = 0.49; 95% CI: 0.27–0.89; *P* = 0.019), Ofx resistance (HR = 0.46; 95% CI: 0.29–0.73; *P* = 0.001), and a sputum smear grade > 1 (HR = 0.61; 95% CI: 0.41–0.91; *P* = 0.001). No significant difference was found between the presence of lung lesions during the baseline chest radiographic examination and sputum culture conversion (HR = 1.17; 95% CI: 0.79–1.71; *P* = 0.32).

**Table 3:** Factors related to sputum culture conversion amongst multidrug-resistant patients in Jiangsu Province, China (*N* = 139)

Variable	N (%)	HR	95% CI	*P*-value
Age, years				
≤ 51	99 (71.4)	Reference		
> 51	40 (28.6)	0.77	0.53–1.14	0.190
Gender				
Male	71 (51.1)	Reference		
Female	68 (48.9)	0.90	0.59–1.36	0.604
Weight, kilograms				
≤ 60	79 (56.8)	Reference		
> 60	60 (43.2)	0.91	0.62–1.33	0.607
Smoking				
No	72 (51.8)	Reference		
Yes	65 (46.8)	0.63	0.43–0.93	0.020
Missing	2 (1.4)			
Drinking				
No	86 (61.9)	Reference		
Yes	26 (18.7)	0.49	0.27–0.89	0.019
Missing	27 (19.4)			
Ofloxacin				
Drug sensitive	103 (74.1)	Reference		
Drug resistance	15 (10.8)	0.46	0.29–0.73	0.001
Missing	21 (15.1)			
Kanamycin				
Drug sensitive	81 (30.2)	Reference		
Drug resistance	42 (58.3)	0.72	0.37–1.38	0.320
Missing	16 (11.5)			
Sputum smear grade				
≤ 1	51 (36.7)	Reference		
> 1	87 (62.6)	0.61	0.41–0.91	0.001
Missing	1 (0.7)			
Lung lesions				
No	3 (2.2)	Reference		
Yes	129 (92.8)	1.17	0.79–1.71	0.436
Missing	7 (5.0)			
Baseline regimens				
SR	77 (55.4)	Reference		
IR	62 (44.6)	1.34	0.76–2.37	0.32

Abbreviations: *HR* hazard ratio, *CI* confidence interval, *IR* individualized regimens, *SR* standard regimen. SR: include SR1, SR2, SR3 and SR4 regiments

We further performed a multivariate regression and found that smoking (HR = 0.44; 95% CI: 0.23–0.83; *P* = 0.011), drinking (HR = 0.41; 95% CI: 0.21–0.81; *P* = 0.011), Ofx resistance (HR = 0.43; 95% CI: 0.24–0.76; *P* = 0.003) and a sputum smear grade > 1 (HR = 0.51; 95% CI: 0.31–0.83; *P* = 0.008) continued to be associated with sputum culture conversion (Table 4).

**Table 4:** Multivariate analysis of predictors of sputum culture conversion among multidrug-resistant patients in Jiangsu Province, China (*N* = 139)

Variable	Adjusted Model*		
	HR	95% CI	*P*-value
Age, years			
≤ 51	Reference		
> 51	1.09	0.62–1.91	0.766
Gender			
Male	Reference		
Female	0.61	0.34–1.11	0.105
Smoking			
No	Reference		
Yes	0.44	0.23–0.83	0.011
Drinking			
No	Reference		
Yes	0.41	0.21–0.81	0.011
Ofloxacin			
Drug sensitive	Reference		
Drug resistance	0.43	0.24–0.76	0.003
Sputum smear grade			
≤ 1	Reference		
> 1	0.51	0.31–0.83	0.008

Abbreviations: *HR* hazard ratio, *CI* confidence interval; *Adjusted for age, gender, alcohol status, smoking status, resistance to Ofloxacin, and sputum smear grade

## Discussion

To our knowledge, there is no similar study in China, to investigate factors that influenced sputum culture conversion in the process of treatment of MDR-TB disease. In this setting with a high burden of drug resistant tuberculosis, we further validated that time to sputum culture conversion is a useful prognostic tool to predict end-of-treatment outcomes in MDR-TB patients but that results differ substantially depending on the month of treatment in which conversion occurs [5, 6]. The median time of sputum smear conversion was 91.5 days, which was similar with a recent study by B. Velayutham and longer than it reported in earlier studies [14–17]. This might because during the time waiting for the DST results, clinicians usually give the anti-TB drugs according to their experience and previous medication history of the patients, which could results in the culture conversion of the patients before the initiation of the standardized treatment, who were then were excluded before the initiation of the treatments.

After multivariable analysis, we further assessed factors associated with increased delay in culture conversion. We found that resistance to Ofx, smoking, drinking, and a high sputum smear grade were risk factors affecting culture conversion. Yuen et al. suggested that MDR-TB regimens including more potentially effective drugs are likely to improve treatment response in MDR-TB patients [18]. To evaluate the association between regimen composition and treatment response, we made a comparison between individualized and standardized drug regimens and treatment outcomes. We found no statistically significant differences however this may be due to statistical power during stratification.

Substantial evidence exists indicating that smoking delays culture conversion and adversely affects end-of-treatment outcomes in drug-susceptible TB patients [19–22]. For example, Maciel et al. found that TB patients that smoked were approximately three times less likely to convert their sputum culture after two months compared to non-smoking TB patients. Leung CC et al. also found that approximately one in six treatment failures were due to patient smoking habits [20]. A recent study conducted in Brazil demonstrated that tobacco smoking delayed culture conversion during treatment for pulmonary tuberculosis and that this relationship was dose-dependent [21]. Whether smoking also delays culture conversion in MDR-TB patients has not been investigated previously and we were able to extend these previous findings in drug-susceptible TB patients [19–21] to MDR-TB patients. In this study, we found that smokers had a higher risk for persistent culture-positivity compared with nonsmokers after adjustment for potential confounders. MDR-TB patients may have ceased smoking after hospital admission and this could have led to misclassification of patient smoking status. However, this bias, if present, likely brings the association between smoking and delayed culture conversion among MDR-TB patients towards the null. Smoking cessation programs in an attempt to reduce poor treatment outcomes may be an important supplementary control measure for TB programs dealing with a high-burden of drug resistance TB, such as China.

A few studies have shown that sputum culture conversion can be delayed in patients who have infecting isolates resistant to second line anti-TB drugs [6, 22, 23]. After adjustment for confounders, we found that participants resistant to Ofx were significantly less likely to have sputum culture conversion. Fluoroquinolones are amongst the most effective medications for patients not susceptible to first line drugs and therefore play an important role in treating drug resistant tuberculosis [24, 25]. Nevertheless, excessive and irregular medication were leading causes for fluoroquinolone resistance in Pakistan [26, 27]. We found that, patients resistant to Ofx were over two times more likely to have continuous culture-positive laboratory tests by the end of multidrug-resistant TB treatment. Fluoroquinolone resistance is a potential critical threat to the control of drug resistant TB globally and efforts to prevent resistance through high medication compliance in high-burden settings are essential.

Rie Kanda et al. reported the time of the sputum culture conversion was prolonged by high smear grade [28], consistent with the results of F. Qazi et al. in 2011 [22] and Caetano Mota et al. in 2012 [29]. Also, patients with a high colony count were less likely to convert than those who had a low smear grade. In our study, patients with a smear grade > + 1 had a lower likelihood of sputum smear conversion compared with those with a smear grade ≤ + 1. It might be natural that MDR-TB patients who had a higher colony count take a longer time for sputum culture conversion. This result highlights the importance of early detection and treatment of drug resistant TB patients.

There are several limitations of this study. First, all patients ceased smoking and drinking during treatment, which may have led to nondifferential misclassification driving our results toward the null. Moreover, we did not quantify smoking or drinking in more detail which may have led to a lack of an effect on culture conversion. Second, missing information was present among some of our variables of interest. To account for this, we attempted to retrieve any missing information by revisiting participants and checking the infectious disease reporting system. Third, this survey did not collect information on HIV infection status and this may potentially be an important confounder. Patients with tuberculosis in China are not routinely tested for HIV and, due to this we were unable to assess the influence of HIV coinfection.

## Conclusions

In conclusion, we present a prospective cohort study of MDR-TB patients from urban China and we found MDR-TB patients that smoke, drink, have ofloxacin resistance, or a high smear grade are less likely to respond to treatment and should be meticulously followed up. A multidimensional approach is needed to effectively control TB, including early detection and treatment, and proper interventions to lower the drinking rate and cigarette smoking rate.

## Abbreviations

AFB: acid-fast bacillus
Am: amikacin
Cm: capreomycin
Cs: cycloserine
DST: traditional drug sensitivity tests
E: ethambutol
HIV: human immunodeficiency virus
HR: hazard ratio
INH: rifampicin
IR: individualized regimens
Km: kanamycin
Lfx: levofloxacin
MDR-TB: multidrug-resistant tuberculosis
Mfx: moxifloxacin
MTB: Mycobacterium tuberculosis
Ofx: ofloxacin
PAS: para-amino salicylic acid
Pto: protionamide
RMP: isoniazid
SR: standard regimen
TB: tuberculosis
Z: pyrazinamide
